# The medical assistance system and inpatient health care provision: Empirical evidence from short-term hospitalizations in Japan

**DOI:** 10.1371/journal.pone.0204798

**Published:** 2018-10-04

**Authors:** Michio Yuda

**Affiliations:** Graduate School of Economics and Management, Tohoku University, Sendai, Miyagi, Japan; TNO, NETHERLANDS

## Abstract

This paper uses two nationally representative sets of medical claims data from medical assistance and universal public health insurance systems to examine how medical assistance system assignment affects short-term inpatient health care provision. In Japan, the medical assistance system, which is part of a public assistance system, provides medical care services for its beneficiaries without imposing any financial burdens, such as copayments or advance premium payments. These circumstances can lead to inpatient costs, as physicians may provide more treatments because there is a financial incentive. Because the assignment of public assistance in Japan is not random but is subject to means testing by the local government, I employ the instrumental variable model to control the potential correlation. I find that medical expenditure is significantly higher for medical assistance patients than for universal public health insurance patients, with an arc elasticity of approximately 0.20. This elasticity is slightly greater than that found for inpatient care in the randomized RAND Health Insurance Experiment and recent empirical studies on low-income populations. In addition, the elasticities for patients who receive medication, treatment and surgery are greater.

## Introduction

Welfare systems guarantee a minimum standard of living and provide independence for those who are destitute based on their level of need. In general, the level of welfare supplied is subject to means testing, and the eligible beneficiaries are typically provided with income security, employment support, and medical and long-term care financed by taxation. Health care services in particular are important in enabling individuals to maintain and improve their health status, quality of life, and life expectancy. As evidence for this statement, Currie et al. [1], Travis [2], Baker and Royalty [3], Choi [4], Gross and Notowidigdo [5], Finkelstein et al. [6], and Chandra et al. [7] find that Medicaid and other health insurance schemes in the US have contributed to improvements in access to health care services, health status, and quality of life for low-income populations.

In Japan, the medical assistance (MA) system, which is part of a public assistance (PA) system, provides medical care services for its beneficiaries without any financial burden for them, and Kumagai [8] and Hayashi [9] find that the MA system has contributed to maintaining and improving the health of the Japanese PA beneficiaries. Specifically, Kumagai [8] examines the MA system efficiency using prefectural data and finds that income transfers from the central government to local governments have contributed to improving the health status of beneficiaries. Hayashi [9] examines the effects of medical factors on transfer deficits in the PA system using municipal government data. The findings indicate that the central grants effectively accommodate changes in local needs. However, Hayashi [9] notes that the number of beneficiary households and factors related to mental illness among the recipients cause a transfer deficit toward the top of the conditional quantile, which compromises regional equity in financing medical care for MA beneficiaries.

The Japanese government is currently considering drastic changes in health care reimbursement for MA beneficiaries because medical care expenses for PA beneficiaries have represented the greatest share of PA expenses since the 1950s, an average of 56.1 percent, which is 1.63 times greater than the average of livelihood assistance expenses. This expansion in MA expenses has been detrimental to the fiscal situation of local governments, which bear one-quarter of the medical costs. One possible major reason for this expansion is that MA patients receive medical treatments at no cost to them; thus, The Government Revitalization Unit [10] and The Fiscal System Council [11] proposed introducing copayments for MA patients and mandating prescriptions of generic drugs. Because the former policy reform would be a particularly drastic environmental change for MA patients, it is necessary to strictly predict the policy effect on MA patients’ health and health care utilization. Although the behaviors of public health insurance patients and medical suppliers increased medical expenditures in Japan [12–19] due to ex post moral hazard and supplier-induced demand (SID), there are not yet any studies or even fundamental empirical studies that investigate health care utilization by MA patients.

In this paper, I use two nationally representative sets of claims data from the Japanese Ministry of Health, Labor and Welfare (MHLW) to compare short-term inpatient health care provision between MA and public health insurance (PHI) patients. The results provide extremely important policy implications for Japanese health policy reform and the PA and MA systems because this study provides the first empirical research on MA patients’ inpatient health care utilization. The RAND Health Insurance Experiment (HIE) has already provided some helpful insights into this issue; its findings indicate that the price elasticity for inpatient treatment is 0.17 [20–22]. However, there are no guarantees that this US result from the 1970s has external validity because health care systems, medical technologies, and disease types in the US at that time were quite different from those in other developing and other developed countries, including Japan, at the present time. In addition, it is necessary to consider the specific socioeconomic characteristics of low-income patients [7], which are not considered in the RAND HIE. Specifically, (i) low-income patients may simply be more price responsive because they face tighter budget constraints; (ii) lower-income individuals may be less able to evaluate the marginal benefit of their care than higher-income individuals and, as a result, may have a higher propensity to reduce high-marginal-benefit care; and (iii) higher rates of chronic illness among low-income populations could imply differential effects of cost-sharing in low-income populations. More recently, Chandra et al. [7] used exogenous variation in the copayments faced by low-income enrollees in the Massachusetts Commonwealth Care Program (MCCP) to examine the impact of patient cost-sharing increases on health care utilization. They found that the price elasticity with respect to hospital spending is estimated as –0.115, which is lower than that of the randomized RAND HIE. However, this result does not necessarily have external validity for the Japanese MA system because MA and other PA benefits are comprehensively provided within the government’s budget. Moreover, there is at least an advantages of examining the Japanese system. Specifically, there is no regional heterogeneity in Japan like that observed in the Medicaid system in the US. Specifically, MA patients can receive exactly the same medical treatment as PHI patients, and physicians can receive the same revenue by providing treatments to MA and PHI patients under the Japanese national fee schedule, independent of the physician’s skill and experience.

In this study, I obtain two main findings. First, MA assignment increases medical expenditure, with an arc elasticity ranging from 0.195 to 0.200. This elasticity is slightly greater than that found for inpatient care in the RAND HIE, in the MCCP, and in recent Japanese studies using only PHI patients. Second, elasticities for patients who receive medication and treatment and operation are more elastic.

## Materials and methods

### Institutional background

The current Japanese PA system, which is one of the oldest social security programs in Japan, has ensured that the Japanese people have the right to maintain a minimum standard of wholesome and cultural living since 1950 [23]. The PA system comprehensively provides its beneficiaries with income security, employment support, and medical and long-term care financed by national and local taxes. The national government contributes 75 percent of the PA expense, and local governments contribute the remaining 25 percent. The level of welfare supplied is subject to means testing by the local prefectural and municipal governments, which comprehensively consider the applicant’s assets, operating capacity, and family support. Generally, city inhabitants submit their PA applications to a municipal welfare office, and inhabitants of towns or villages submit applications to a prefectural welfare office.

The MA system, which is part of the PA system, provides medical care services for its beneficiaries but has several institutional differences from the universal PHI system. S1 Table summarizes the differences between the MA and PHI systems in Japan. Although universal PHI subscribers pay advance premiums, copayments and taxes to receive medical care, MA patients receive exactly the same medical coverage as PHI patients without these financial burdens; public funds fully finance the beneficiaries’ medical costs under the Japanese MA system.

These circumstances are likely to account for two major inefficiencies in health economics, namely, excess utilization by the beneficiaries (ex post moral hazard) and excess medical care provision by the medical suppliers (the physician agency problem), which could significantly increase medical costs. Regarding inpatient treatment, the latter problem in particular may lead to more costs, as physicians may provide more treatments because of the financial incentive, known as SID, because they know that MA patients can receive treatments without any need for copayment. The most extreme case occurred in Yamamoto Hospital in 2009. The hospital director was arrested for medical billing fraud involving fictitious treatments, professional negligence, and the involuntary manslaughter of several MA patients due to irrelevant and unprofessional surgical operations. Another reason that MA expenses account for such a large share of public expenses is that welfare recipients initially have a great need for health care. A 2012 survey by the MHLW reported that approximately 75 percent of households benefiting from the Japanese PA system included aged (48.1%), injured or diseased (16.1%), or disabled (11.1%) people. This patient structure itself may result in a significant increase in medical expenditure for MA patients.

### Data

I use two nationally representative sets of individual-level data for the period 2000–2010, namely, the *Fact-finding Survey on Medical Assistance* (*Iryo-Fujo Jittai Chosa*) and the *Survey of Medical Care Activities in Public Health Insurance* (*Syakai Iryo Shinryo-Koui Betsu Chosa*). The MHLW conducted these surveys to obtain basic information for system administration and to provide claims data for the reimbursement of medical care costs for both MA and PHI patients. Both are repeated cross-sectional surveys consisting of randomly selected claims data assessed in June each year. The data in common include each patient’s gender, age, residential prefecture, monthly medical expenditure, actual days of care, main classified illness according to the International Statistical Classification of Disease and Related Health Problems (ICD-10), duration of medical facility visits, and type of medical institution (hospital or clinic).

The sample used in this study consists of patients admitted to a medical institution for a length of stay not exceeding 31 days in May each year because using repeated cross-sectional data for a certain month in a year is not appropriate for analyses that track the course of a patient’s disease.

In other words, these data are useful to estimate the correct effect of MA assignment on inpatient health care provision in the short term because short-term hospitalization has a high likelihood of representing a complete episode. In addition, the medical expenditure of the patients in the sample is reimbursed through the fee-for-service payment scheme because information on PHI patients participating in the prospective payment system was unavailable. However, because claims data have some disadvantages, as do most discharge data, the results of this study should be carefully interpreted. First, as with the data used in Chandra et al. [7], Shigeoka [18], and Fukushima et al. [19], they include only limited individual characteristics, such as age, gender, and place of residence. There are no records for education, income, and health conditions in either survey, and admission health outcomes, such as rehospitalization and death, are not included in the *Fact-finding Survey on Medical Assistance*. Second, they do not include non-users, as with Shigeoka [18]. Therefore, the estimated parameters may be lower than those that include non-users (healthy people) because non-users are generally more price sensitive than health care consumers. However, Shigeoka’s [18] estimated price elasticity based on repeated cross-sectional surveys is similar to that of another paper on the same issue, Fukushima et al. [19], which uses panel data including patients and non-users.

Table 1 presents descriptive statistics for the sample and mean comparison tests. The sample consists of 18,693 MA and 280,827 PHI patients. The mean medical expenditure is 77,741 yen greater for MA than for PHI inpatients. In addition, MA patients are on average 10.1 years older than PHI patients, but the proportion of MA female patients is 4.5 percent lower. Moreover, the proportions of MA patients with the following main illnesses are significantly higher than those of PHI patients: mental and behavioral disorders (7.9 percentage points (pp)); diseases of the circulatory system (6.5 pp); and endocrine, nutritional, and metabolic diseases (4.8 pp). Conversely, the proportions of MA patients with the following main illnesses are significantly lower than those of PHI patients: pregnancy, childbirth, and puerperium (8.4 pp); diseases of the eye and adnexa (6.8 pp); and diseases of the genitourinary system (4.7 pp). The number of hospital visits is also 42.1 pp higher for MA patients than for PHI patients.

**Table 1:** Descriptive statistics.

Sample	All patients		MA patients		PHI patients			
Variables	Mean	Std. Dev.	Mean	Std. Dev.	Mean	Std. Dev.	Mean Difference^c^	Std. Err.
Dependent variable								
Monthly medical expenditure (thousand yen in 2010 prices)	230.03	266.77	302.92	313.11	225.17	262.68	77.74**^c^	2.34
Independent variable								
Medical assistance (= 1)	0.06	0.24	1.00	0.00	0.00	0.00		
Gender (female = 1)	0.52	0.50	0.48	0.50	0.53	0.50	-0.05**^c^	0.00
Age ^a^	53.71	25.64	63.16	18.42	53.08	25.92	10.09**^c^	0.14
Predominant diseases (ICD-10, large classifications) ^b^								
Certain infectious and parasitic diseases (= 1)	0.04	0.19	0.04	0.19	0.04	0.19	0.00	0.00
Neoplasms (= 1)	0.10	0.30	0.10	0.29	0.10	0.30	-0.01**^c^	0.00
Diseases of the blood and blood-forming organs and certain disorders involving the immune mechanism (= 1)	0.00	0.06	0.00	0.07	0.00	0.06	0.00	0.00
Endocrine, nutritional and metabolic diseases (= 1)	0.04	0.19	0.08	0.28	0.03	0.18	0.05**^c^	0.00
Mental and behavioral disorders (= 1)	0.03	0.16	0.10	0.30	0.02	0.15	0.08**^c^	0.00
Diseases of the nervous system (= 1)	0.03	0.17	0.04	0.18	0.03	0.17	0.00**^c^	0.00
Diseases of the eye and adnexa (= 1)	0.10	0.30	0.04	0.19	0.11	0.31	-0.07**^c^	0.00
Diseases of the ear and mastoid process (= 1)	0.01	0.08	0.00	0.06	0.01	0.08	0.00**^c^	0.00
Diseases of the circulatory system (= 1)	0.13	0.34	0.20	0.40	0.13	0.34	0.07**^c^	0.00
Diseases of the respiratory system (= 1)	0.11	0.31	0.08	0.28	0.11	0.31	-0.02**^c^	0.00
Diseases of the digestive system (= 1)	0.09	0.29	0.10	0.30	0.09	0.29	0.01**^c^	0.00
Diseases of the skin and subcutaneous tissue (= 1)	0.01	0.08	0.01	0.08	0.01	0.08	0.00	0.00
Diseases of the musculoskeletal system and connective tissue (= 1)	0.04	0.21	0.06	0.24	0.04	0.20	0.02**^c^	0.00
Diseases of the genitourinary system (= 1)	0.09	0.28	0.04	0.20	0.09	0.29	-0.05**^c^	0.00
Pregnancy, childbirth and the puerperium (= 1)	0.08	0.28	0.01	0.08	0.09	0.29	-0.08**^c^	0.00
Certain conditions originating in the perinatal period (= 1)	0.01	0.12	0.00	0.04	0.01	0.12	-0.01**^c^	0.00
Congenital malformations, deformations and chromosomal abnormalities (= 1)	0.00	0.06	0.00	0.04	0.00	0.06	0.00**^c^	0.00
Symptoms, signs and abnormal clinical and laboratory findings, not elsewhere classified (= 1)	0.02	0.14	0.03	0.16	0.02	0.14	0.01**^c^	0.00
Injury, poisoning and certain other consequences of external causes (= 1)	0.06	0.24	0.08	0.27	0.06	0.24	0.02**^c^	0.00
Medical supplier's characteristics								
Hospital (= 1)	0.44	0.50	0.84	0.37	0.41	0.49	0.42**^c^	0.00
Regional Characteristics								
ln (Private insurance prevalence)^d^	0.36	0.83	0.56	1.00	0.35	0.82	0.21**^c^	0.01
ln (Unemployment rate)^e^	1.52	0.23	1.58	0.23	1.52	0.23	0.06**^c^	0.00
ln (Physician density)^f^	2.27	0.19	2.30	0.22	2.27	0.19	0.03**^c^	0.00
ln (Nurse density)^g^	3.96	0.08	3.97	0.09	3.96	0.08	0.01**^c^	0.00
ln (Share of hospital beds of public hospital)^h^	3.23	0.37	3.17	0.33	3.24	0.37	-0.06**^c^	0.00
ln (Public assistance expenditure)^i^	2.90	0.58	3.19	0.53	2.88	0.57	0.31**^c^	0.00
Number of observations	299,520		18,693		280,827			

^a^ The age of patients aged 100 and over is set as 99 because of the original data specification. In the empirical analyses, the reference group is patients aged zero.

^b^ In the empirical analyses, 119 middle-classified predominant diseases according to the ICD-10 are used, and the reference group is the disease described as “symptoms, signs and abnormal clinical and laboratory findings, not classified elsewhere”.

^c^ ** represents statistical significance at the 1percent level.

^d^ Sourced from *Life Insurance Fact Book*, the Life Insurance Association of Japan.

^e^ Sourced from *Labour Force Survey*, Statistics Bureau, the Ministry of Internal Affairs and Communications.

^f^ Sourced from *Survey of Physicians*, *Dentists and Pharmacists*, the Ministry of Health, Labour, and Welfare.

^g^ Sourced from *Survey of Medical Institutions*, the Ministry of Health, Labour, and Welfare.

^h^ Sourced from *Report on Public Health Administration and Services*, the Ministry of Health, Labour, and Welfare.

^i^ Source from *Survey on Local Public Finance Conditions*, the Ministry of Internal Affairs and Communications.

### Identification strategy

#### The basic model

To estimate the effect of MA assignment on medical expenditure, I specify the following simple linear regression model:

$$Y_{it}=\alpha_{0}+\alpha_{MA}{MA}_{it}+x_{it}\alpha +\lambda +\tau_{t}+u_{it},$$ (1)

where *Y*_*it*_ is patient *i’*s medical expenditure adjusted to 2010 prices using the consumer price index (CPI) in year *t*. *MA*_*it*_ is a dummy variable representing assignment under the MA system such that *MA*_*it*_ = 1 if patient *i* is an MA patient and *MA*_*it*_ = 0 if patient *i* is a PHI patient. In fact, although the copayment rate differs by age and income level among the PHI subscribers, it cannot be correctly identified because of limited information on patients in claims data. The parameter *α*_*MA*_ represents the average treatment effect (ATE) and is significantly positive if a physician provides an MA patient with more inpatient treatment than a comparable PHI patient.

The vector **x**_**it**_ consists of independent variables composed of patients’ and medical suppliers’ characteristics. The patient characteristics include dummy variables for gender (female = 1), age, and 113 types of main illnesses. The medical supplier characteristics include a hospital dummy variable that captures differences in the number and quality of medical devices and the difference in the number of medical staff in hospitals and clinics. Under the Japanese Medical Care Act, the definition of a *hospital* is a medical institution with 20 or more hospital beds. Therefore, some patients are hospitalized in a clinic, which is defined as a medical institution with 19 or fewer hospital beds. The variables *λ* and *τ*_*t*_ represent local (prefectural) and yearly fixed effects, respectively, and *u*_*it*_ is an error term.

#### MA assignment and dummy endogenous variable models

A consistent ATE for *α*_*MA*_ can be obtained by using ordinary least squares (OLS) estimation when the exogenous assumption for *u*_*it*_ holds. However, PA assignment is not random but is subject to means testing by local prefectural and municipal governments. In fact, Suzuki and Zhou [24] and Hayashi [25] find that municipality fiscal status and local socioeconomic conditions lead to significant variations in the local public assistance ratio. In addition, as discussed previously, approximately 75 percent of welfare recipients initially tend to have a great need for health care, and the stigma associated with being a beneficiary may make low-income people hesitant to apply to the PA system. However, the beneficiary may dare to receive low-intensity medical care services to avoid stoppage of PA benefits due to health improvement. This evidence suggests that there are a potentially positive and negative correlations between *MA*_*it*_ and *u*_*it*_, implying bias in the OLS estimators of Eq (1).

To control for this potential correlation, I employ a dummy endogenous variable model [26, 27]. In this study, the following MA assignment equation is first estimated by a probit model to obtain its predicted probability of $\hat{P_{it}}$:

$$Prob({MA}_{it}=1|x_{it},z_{it})=\Phi (\beta_{o}+x_{it}\beta_{x}+z_{it}\beta_{z}+\lambda +\tau_{t}),$$ (2)

where **Φ**(∙) represents the cumulative standard normal distribution and z_it_ is an exogenous variable correlated with PA assignment and unrelated to *u*_*it*_. Next, using $\hat{P_{it}}$ as an instrumental variable for *MA*, Eqs (1) and (3) are estimated using the regular instrumental variable (IV) method:

$${MA}_{it}=\gamma_{0}+x_{it}\gamma_{x}+\gamma_{p}∙\hat{P_{it}}+\lambda +\tau_{t}+e_{it},$$ (3)

where *e*_*it*_ is an error term that is assumed to be exogenous. Using nonlinear prediction as an instrument has the advantage that the resulting two-stage least squares estimates will be more efficient than those found using a linear first-stage regression because the nonlinear model gives a better approximation of the first-stage conditional expected function than the linear model [28].

In addition, the structure of Eq (2) indicates that the predicted probability $\hat{P_{it}}$ includes comprehensive information on medical assistance assignment, as discussed in Suzuki and Zhou [24] and Hayashi [25]. In that sense, it is desirable that z, which is individual patient characteristics or exogenous environment changes, is strongly correlated with PA assignment and unrelated to *u*_*it*_. Unfortunately, because the claims data contain little socioeconomic information for patients in **x**_**it**_, *λ* and *τ*_*t*_, instead I employ the logarithm of the lagged prefectural amount of local governments’ PA expenses adjusted to 2010 prices. The total PA expense depends on the number of beneficiaries and on the actual content of PA support, and it is considered to include comprehensive information on the PA program in each region. According to Suzuki and Zhou [24], local governments with high PA expenses may face fiscal hardship and thus may become strict in making PA assignments. In this case, *MA* and *u* are negatively correlated in Eq (1). However, because most of the PA expenses financed by a local government are subsidized by the local allocation grant [25], local governments may be tolerant of accepting the beneficiaries. In this case, *MA* and *u* are positively correlated. In addition, the PA expenses in the previous year are not considered to influence individual decision making in terms of health care supply in year *t*. These facts indicate that the logarithm of the lagged PA expense satisfies appropriate characteristics in this study. Moreover, its means and standard deviations during the study sample expands (S1 Fig), and these large geographic and time variations, as used in Finkelstein [29], Choi [4], and Kondo and Shigeoka [16], are useful for identifying its effect. In addition, there is a possibility that the estimated parameter is not interpreted as a price effect when a PHI patient’s private insurance mostly covers inpatient medical expenditure. In fact, the *Nationwide Survey for Life Insurance* by the Japan Institute of Life Insurance reports that the private insurance prevalence rate during the study period exceeds 70 percent. As mentioned above, because the claims data contain little socioeconomic information for patients, I add the prefectural variable of private life insurance policies in force per capita, a proxy for private insurance prevalence, as the second best. However, it should be noted that most private health insurance companies in Japan do not reimburse individual copayments but rather provide fixed return insurance per day (Kawaguchi [30]). That is, the insurance plan functions not as risk protection but as income security insurance, such as compensation benefits for temporary disability. In addition, the public health insurance plans financed by insurance premiums and taxation provide most of the medical benefit coverage. Therefore, the prevalence of private health insurance may have little effect on inpatient utilization. Moreover, it should be noted that several recent empirical studies have found that macroeconomic conditions significantly affect both PA assignment and health care utilization. One such factor is business cycle influence. The PA rates generally increase during periods of economic depression; thus, the business cycle significantly affects the population’s health and health care utilization in several developed countries [31–36]. Another factor is the local government’s budgetary condition. Local governments facing fiscal hardship may not only become strict in making PA assignments [24] but may also reduce resources at public medical institutions, such as staff and medical equipment. As these factors may affect both MA assignment and health care utilization, omitting these variables from regression equations leads to bias in the estimated parameters. In this regard, because Chandra et al. [7] do not consider these effects, their estimated parameters may be biased. For example, if increases in the copayment rates and premiums in the MCCP are due to budget difficulties in the Commonwealth of Massachusetts, several changes in medical resources may occur there during the same period. This means that the variable of copayment rate and the error term are correlated. To eliminate this omitted variable bias, I add four prefectural variables as proxies for the two factors of prefectural regional unemployment rate and regional medical resources. The prefectural regional unemployment rate is the proxy for the business cycle, and the proxies for regional medical resources are the number of physicians per 100,000 inhabitants (physician density), the number of nurses per 100,000 inhabitants (nurse density), and the ratio of the number of hospital beds in public medical institutions to the total (public hospital bed ratio). Although some aspects of these factors are already captured by the prefectural fixed effect *λ*, *λ* captures only the time-invariant unobserved heterogeneity and does not contain the remaining time-variant factors. Therefore, adding prefectural heterogeneity is equivalent to considering time-variant regional specific time trends (*μ*_*t*_), and the empirical equations are corrected as follows.

$$Y_{it}=\alpha_{0}+\alpha_{MA}{MA}_{it}+x_{it}\alpha +\lambda +\mu_{t}+\tau_{t}+u_{it}$$ (4)

$${MA}_{it}=\gamma_{0}+x_{it}\gamma_{x}+\gamma_{p}∙\hat{P_{it}}+\lambda +\mu_{t}+\tau_{t}+e_{it}$$ (5)

$\hat{P_{it}}$ in Eq (5) is the probit model’s predicted probability of Eq (6), and S2 Table shows the selected estimation results of MA assignment equations:

$$Prob({MA}_{it}=1|x_{it},z_{it},\lambda ,\lambda_{t},\tau_{t})=\Phi (\beta_{o}+x_{it}\beta_{x}+z_{it}\beta_{z}+\lambda +\mu_{t}+\tau_{t})$$ (6)

In addition, I estimate clustering robust standard errors that allow for correlated residuals within prefectures to consider any serial correlation.

## Results

### Aggregate effect

Table 2 shows the estimated arc elasticity *ε* with respect to the effect of MA assignment on medical expenditures, which is the same formula used in the RAND HIE [37]:

$$\varepsilon =\frac{dy}{dMA}∙\left(\frac{\frac{1({MA}_{it}=1)+1({MA}_{it}=0)}{2}}{\frac{\bar{Y_{MA}}+\bar{Y_{PHI}}}{2}}\right)=\hat{\alpha_{MA}}∙\left(\frac{1}{\bar{Y_{MA}}+\bar{Y_{PHI}}}\right)$$ (7)

where **1**(*MA*_*it*_) is an indicator function for MA assignment, and $\bar{Y_{MA}}$ and $\bar{Y_{PHI}}$ are the sample means for MA and PHI patients, respectively.

**Table 2:** The effect of medical assistance assignment on inpatient health care provision.

Estimation method	OLS^a^^,^ ^b^^,^ ^c^			IV^a^^,^ ^b^^,^ ^c^		
Medical assistance (N = 299,520)	-0.004	-0.004	-0.004	0.200**^cc^	0.198**^cc^	0.195**^cc^
	(0.017)	(0.017)	(0.017)	(0.057)	(0.058)	(0.057)
Adjusted R^2^	0.176	0.176	0.176	0.168	0.168	0.168
First-stage F statistics				1393.520**^cc^	1541.220**^cc^	1578.310**^cc^
Private insurance prevalence	No	Yes	Yes	No	Yes	Yes
Regional specific time trends	No	No	Yes	No	No	Yes

^a^ All equations include a constant term and dummy variables for gender, age, predominant diseases, hospital, area, and year.

^b^ Upper values are estimated arc elasticities, and clustering robust standard errors allowing for correlated residuals within prefectures are in parentheses.

^c^ ** and * represent statistical significance at the 1 and 5 percent levels.

The arc elasticity obtained from the OLS estimation is approximately −0.004, and that of the IV estimation ranges from 0.195 to 0.200. Because the first-stage F-statistics indicate that $\hat{P_{it}}$ has sufficient power to explain PA assignment in the IV models, the IV estimation eliminates the negative bias in the OLS estimators. These results also indicate the possibility that fiscal measures of the local allocation grant affect MA assignment. In addition, they are more elastic than the 0.17 observed for inpatient care in the randomized RAND HIE and the 0.16 indicated in the MCCP and the recent Japanese empirical studies of Shigeoka [18] and Fukushima et al. [19]. These low elasticities with respect to medical expenditure imply that the SID effect on MA patients exists but has little impact on medical expenditure. Moreover, the coefficients of the private insurance prevalence were not significant in all of the models, and the results were largely unchanged.

### Heterogeneity by specific health conditions

Short-term inpatients generally suffer from acute diseases, and the medical care they receive is different from that provided to inpatients with chronic diseases. In this subsection, I investigate how the estimates change for a sample of inpatients with acute diseases, which are defined based on the definition used by Card et al. [38], and those with chronic diseases, which are defined based on the definition used by Goldman et al. [39] and Fukushima et al. [19]. In addition, it has been found that intensive medical care provision to patients before death results in an extremely high medical expenditure in a short period [40–50]. These results indicate that a few patients with ultraexpensive medical expenditure, who have fatal conditions and no choice in whether to receive medical care, could greatly affect the basic results. Ideally, the decedents would be excluded from the sample, but they cannot be identified in the data. Therefore, I additionally investigate how the results are influenced by excluding patients with expenditure in the top 1 percentile (more than 1,313,345 yen) from the original sample.

Table 3 summarizes the empirical results and shows that MA assignment does not have a significant effect on inpatients with acute and chronic diseases. This means that physicians provide the same treatment to MA and PHI patients with these diseases. In addition, MA assignment for non-ultraexpensive patients also has a significantly positive effect on monthly medical expenditure, but its elasticity, ranging from 0.089 to 0.095, is less than that of the aggregate effect. This finding indicates that there are few differences in inpatient treatment provided to MA and PHI patients.

**Table 3:** Estimated arc elasticities by specific health conditions.

Estimation method	OLS^a^^,^ ^b^^,^ ^c^			IV^a^^,^ ^b^^,^ ^c^		
Acute diseases (N = 24,413)	-0.004	-0.004	-0.003	0.185	0.201	0.199
	(0.024)	(0.024)	(0.023)	(0.133)	(0.141)	(0.127)
Adjusted R2	0.091	0.091	0.092	0.083	0.081	0.083
First-stage F statistics				662.053**s3	657.967**s3	709.879**s3
Chronic diseases (N = 49,953)	-0.016	-0.016	-0.016	0.051	0.053	0.050
	(0.019)	(0.019)	(0.018)	(0.043)	(0.043)	(0.043)
Adjusted R2	0.158	0.158	0.158	0.157	0.157	0.157
First-stage F statistics				715.973**s3	854.023**s3	846.008**s3
Excluding the top 1 percentile	0.010	0.009	0.009	0.095*	0.092*	0.089
(N = 296,525)	(0.013)	(0.013)	(0.013)	(0.046)	(0.046)	(0.046)
Adjusted R2	0.220	0.220	0.220	0.218	0.218	0.218
First-stage F statistics				1553.490**s3	1737.370**s3	1778.520**s3
Private insurance prevalence	No	Yes	Yes	No	Yes	Yes
Regional specific time trends	No	No	Yes	No	No	Yes

^a^ All equations include a constant term and dummy variables for gender, age, predominant diseases, hospital, area, and year.

^b^ Upper values are estimated arc elasticities, and clustering robust standard errors allowing for correlated residuals within prefectures are in parentheses.

^c^ ** and * represent statistical significance at the 1 and 5 percent levels.

### Heterogeneity by treatment type

Because a physician decides the type and volume of inpatient treatments for patients, some physicians may be influenced by the financial incentive to provide more profitable treatments to their patients. In this subsection, I investigate how MA assignment affects several types of inpatient treatment provision: diagnostic imaging, examination, medication, and treatment and operation.

Table 4 summarizes the empirical results and shows that the estimated elasticity varies by the type of medical treatment provision. Specifically, the elasticities are elastic for treatment and operation (0.384 to 0.389) and medication (0.362 to 0.367) but inelastic for examination (0.075 to 0.078). On the other hand, MA assignment was not found to have a significant effect on diagnostic imaging.

**Table 4:** Estimated arc elasticities by treatment type.

Estimation method	OLS^a^^,^ ^b^^,^ ^c^			IV^a^^,^ ^b^^,^ ^c^		
Diagnostic imaging (N = 161,179)	-0.054**l3	-0.053**l3	-0.053**l3	0.129	0.133	0.133
	(0.011)	(0.012)	(0.012)	(0.078)	(0.078)	(0.080)
Adjusted R2	0.155	0.155	0.155	0.150	0.149	0.149
First-stage F statistics				704.549**l3	797.835**l3	822.030**l3
Examination (N = 264,183)	-0.046**l3	-0.046**l3	-0.046**l3	0.078*	0.075*	0.075*
	(0.007)	(0.007)	(0.007)	(0.031)	(0.030)	(0.029)
Adjusted R2	0.273	0.273	0.273	0.271	0.271	0.271
First-stage F statistics				1095.820**l3	1159.500**l3	1192.590**l3
Medication (N = 242,457)	0.127**l3	0.128**l3	0.128**l3	0.362**l3	0.367**l3	0.364**l3
	(0.015)	(0.015)	(0.015)	(0.059)	(0.057)	(0.056)
Adjusted R2	0.082	0.083	0.083	0.076	0.076	0.076
First-stage F statistics				1053.520**l3	1123.750**l3	1154.500**l3
Treatment and operation (N = 173,002)	0.098**l3	0.098**l3	0.098**l3	0.389**l3	0.389**l3	0.384**l3
	(0.023)	(0.022)	(0.022)	(0.075)	(0.080)	(0.078)
Adjusted R2	0.162	0.162	0.162	0.157	0.157	0.157
First-stage F statistics				921.361**l3	956.140**l3	992.673**l3
Private insurance prevalence	No	Yes	Yes	No	Yes	Yes
Regional specific time trends	No	No	Yes	No	No	Yes

^a^ All equations include a constant term and dummy variables for gender, age, predominant diseases, hospital, area, and year.

^b^ Upper values are estimated arc elasticities, and clustering robust standard errors allowing for correlated residuals within prefectures are in parentheses.

^c^ ** and * represent statistical significance at the 1 and 5 percent levels.

## Discussion

In this paper, I use two nationally representative sets of medical claims data from the MHLW in Japan to examine how assignment to the MA system affects the provision of short-term inpatient health care for MA beneficiaries. I find that MA assignment generally increases monthly medical expenditure, with an arc elasticity ranging from 0.195 to 0.200. This elasticity is slightly greater than that found for inpatient care in the randomized RAND HIE, in the MCCP, and in recent Japanese studies using only PHI patients. Second, the significant elasticity varies by the type of medical services provided and patients’ health conditions, ranging from 0.075 to 0.389.

The finding of low elasticities with respect to medical expenditure implies that health care provision to MA beneficiaries would not change drastically even if the government introduced a low fixed copayment or a low copayment rate. Therefore, the efficiency of the MA system as well as the fiscal status of local governments could be improved without worsening the health of MA beneficiaries. In addition, the findings of this study imply that the result indicating that low-income patients are price insensitive is robust despite institutional differences among the unitary and composite systems, such as Medicaid in the US and the Japanese MA system.

This study has some limitations. First, the claims data do not include detailed individual heterogeneity, such as education, income, life habits, family structure, and disease history and severity, or potentially useful characteristics of medical suppliers, such as number of staff and beds, available medical equipment, and type of medical agency. In particular, information about patients’ health conditions in the past and just before the hospitalization may have a strong effect on MA patients’ medical expenditures or on may make their threshold for hospitalization lower. Thus, there is a potential bias in the estimates if there is a correlation between these factors and the independent variables in the equations, especially when using repeated cross-sectional data. In particular, the lack of income information may cause an estimation bias. Because it is generally known that health and income are positively correlated (Currie [51]), the MA dummy variable in Eqs (1) and (4) includes the effects of both the difference in the copayment rate and the health capital stock. For example, because MA patients are generally poorer in health than PHI patients, the difference between them in health at admission may lead to a major difference in medical expenditure. Unfortunately, health status is also unavailable in the data, and it is highly likely that a potential bias in the estimates remains. If health at admission is the same for MA and PHI patients, one good way to separate these effects is to employ the subsample of patients aged 3 and under, because the copayment for PHI patients aged 3 and under has been fully covered by prefectural public subsidy since 1994 [52, 53]. This means the major difference between MA and PHI patients is the health capital stock based on family income level because the actual copayment of any patient aged 3 and under is zero. However, to my knowledge, no empirical studies have found that health at admission is the same for both MA and PHI patients in Japan. To this limitation, the claims data from the diagnosis procedure combination/ per-diem payment system (DPC/PDPS) may be a useful dataset to determine a patient’s health condition at the beginning of admission. However, the DPC/PDPS is generally applied to the patients with acute illness, but those patients tend to have no choice about receiving medical cares despite the presence or absence of the copayments. Second, the claims data used do not include information on patient outcomes. Thus, it was not possible to evaluate how the MA system directly influences the utility of MA beneficiaries and social welfare. Third, because the DPC/PDPS is the prospective payment system, constant medical fees are reimbursed to the medical institutions in spite of the presence or absence of the copayments, which does not provide a financial incentive to the medical suppliers. In addition, because the data in this study are from a repeated cross-section during a certain month in each year, it is impossible to consider individual-level variations in health and health care utilization over time. Finally, information regarding the reason for being a PA beneficiary, the monthly benefit amount, and other related attributes of beneficiaries could improve the estimates. Further analysis using other nationally representative and comprehensive data that contain more detailed variables would help to improve and confirm my findings.

## Supporting information

S1 Fig Within and between variations in the PA expenditure. (TIF)

S1 File Supporting information on “The medical assistance system and inpatient health care provision: Empirical evidence from short-term hospitalizations in Japan”. (DOCX)

S2 File References. (DOCX)

S1 Table Institutional comparison between the medical assistance and public health insurance systems in Japan. (DOCX)

S2 Table Estimation results of the MA assignment equation. (DOCX)
