# Supplementary figures and images for: The medical assistance system and inpatient health care provision: Empirical evidence from short-term hospitalizations in Japan
Source: PLoS One. 2018 Oct 4;13(10):e0204798. doi: 10.1371/journal.pone.0204798 (PMC6171890; doi:10.1371/journal.pone.0204798)

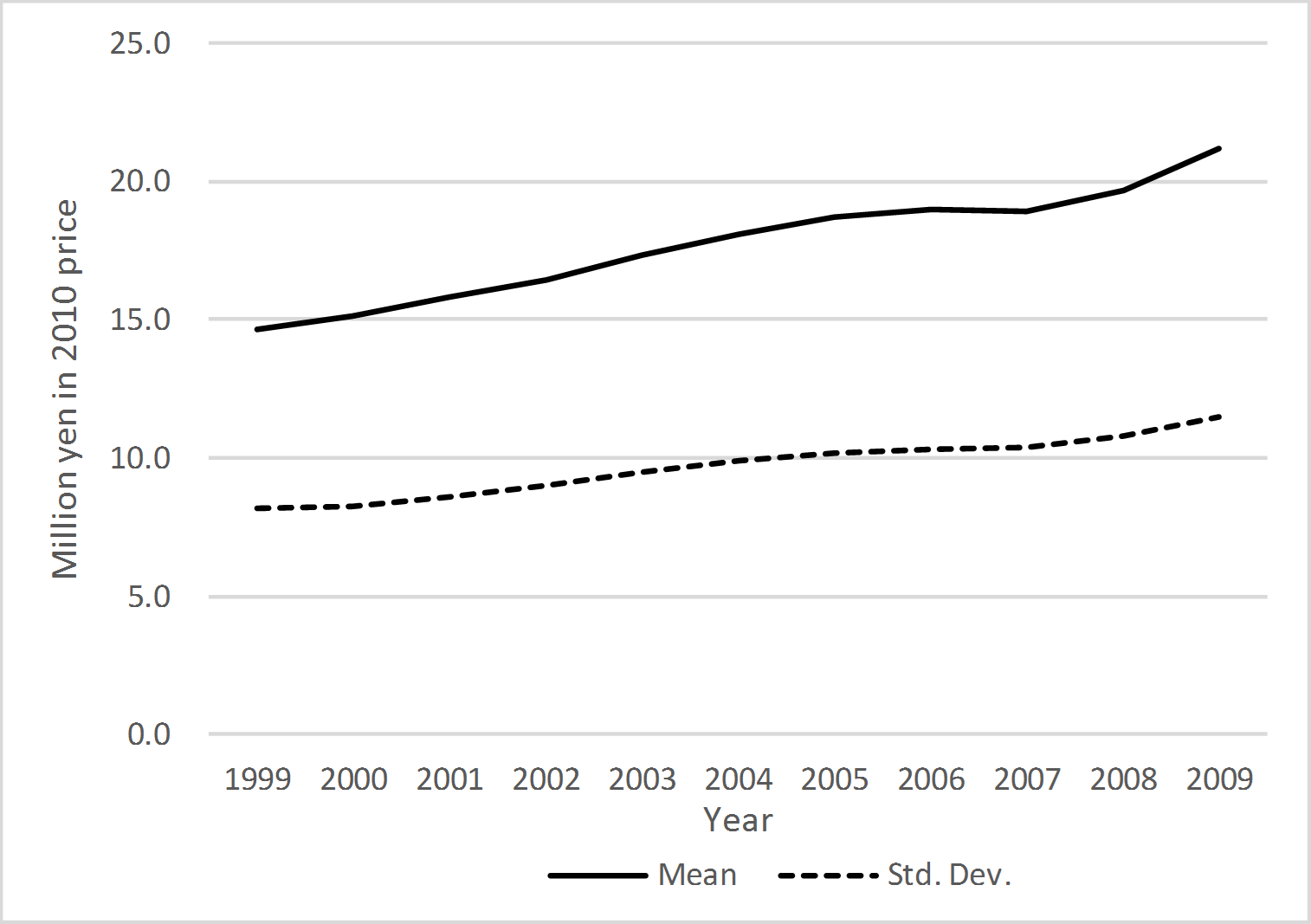

Supplement: S1 Fig — (TIF) [file pone.0204798.s001.tif]
