# Supplementary material for: The medical assistance system and inpatient health care provision: Empirical evidence from short-term hospitalizations in Japan
Source: PLoS One. 2018 Oct 4;13(10):e0204798. doi: 10.1371/journal.pone.0204798 (PMC6171890; doi:10.1371/journal.pone.0204798)
Supplement: S1 File — (DOCX) [file pone.0204798.s002.docx]

Supporting information on “The medical assistance system and inpatient health care provision: Empirical evidence from short-term hospitalizations in Japan”

Appendix A. Institutional comparison between the medical assistance and public health insurance systems in Japan

S1 Table summarizes a comparison of the medical assistance and public health insurance systems in Japan.

Appendix B. Within and between variations in the PA expenditure

S1 Fig summarizes within and between variations in the PA expenditure and shows that the mean and standard deviation increase by 44.4 percent and 40.7 percent, respectively, during the study period. These trends reveal that PA expenditure has large geographic and time variations.

Appendix C. Selected estimation results of the MA assignment equation

S2 Table summarizes the selected estimation results of the MA assignment equation.
