# Supplementary material for: The medical assistance system and inpatient health care provision: Empirical evidence from short-term hospitalizations in Japan
Source: PLoS One. 2018 Oct 4;13(10):e0204798. doi: 10.1371/journal.pone.0204798 (PMC6171890; doi:10.1371/journal.pone.0204798)
Supplement: S2 File — (DOCX) [file pone.0204798.s003.docx]

**References**

S1. Suzuki W. Medical care and the social assistance system. In: Abe A, Kunieda S, Suzuki W, Hayashi M, editors. Economic analyses on the social assistance system. Tokyo: The University of Tokyo Press; 2008. pp. 147-171.
