# Supplementary material for: The medical assistance system and inpatient health care provision: Empirical evidence from short-term hospitalizations in Japan
Source: PLoS One. 2018 Oct 4;13(10):e0204798. doi: 10.1371/journal.pone.0204798 (PMC6171890; doi:10.1371/journal.pone.0204798)
Supplement: S1 Table — (DOCX) [file pone.0204798.s004.docx]

**S1 Table. Institutional comparison between the medical assistance and public health insurance systems in Japan.**

|  | Medical assistance system | Public health insurance |
| --- | --- | --- |
| Financial source | Public funds (100%) | Premiums, copayments, and public funds |
| Copayment rate | 0% | 10% for those aged 75 and over^a^, 20% for preschool children and those aged between 70 and 74^b^, and 30% for all other enrollees^c^ |
| Patient access control | Patient must obtain authorization for medical care and drug tickets | None (free-access system) |
| Coverage | As for public health insurance | In-kind (90%) and cash benefit (10%) |
| Medical supply | Designated medical facilities under the *Public Assistance Act* | Designated medical facilities under the *Health Insurance Act* and the *National Health Insurance Act*. |
| Medical fee schedule^d^ | As for public health insurance | Nationally uniform medical fee schedule |
| Review of claims | Municipalities or local welfare offices | The Social Insurance Medical Fee Payment Foundation and the Federation of National Health Insurance Organizations. |
| Population share (in 2014) | 1.7% | 98.3% (universal health insurance) |

^a^ In total, 30 percent for the persons aged 75 and over with more than a certain income.

^b^ Due to the preferential measure, 10 percent for those aged between 70 and 74 since April 2008. In addition, 30 percent for the persons aged 75 and over with more than a certain income.

^c^ When the patient’s copayment is too high, the difference between the total monthly copayment and the upper limit, depending on the insured person’s age and income level, is repaid by the *high-cost medical treatment system*.

^d^ The fee schedule is reviewed biannually by the Central Social Insurance Medical Council, consisting of insurer, physician and intellectual representatives. Regardless of physician age, experience, position, and skill or individual patient attributes, the medical fees reimbursed for treatment are identical for MA and PHI patients.

^e^ Adapted from Suzuki [S1].
