# Supplementary material for: The medical assistance system and inpatient health care provision: Empirical evidence from short-term hospitalizations in Japan
Source: PLoS One. 2018 Oct 4;13(10):e0204798. doi: 10.1371/journal.pone.0204798 (PMC6171890; doi:10.1371/journal.pone.0204798)
Supplement: S2 Table — (DOCX) [file pone.0204798.s005.docx]

**S2 Table. Estimation results of the MA assignment equation.**

| Model^a, b, c^ | Coef/ SE | Coef/ SE | Coef/ SE |
| --- | --- | --- | --- |
| ln (Public assistance expenditure) | 1.121** | 1.141** | 1.114** |
|  | (0.265) | (0.241) | (0.240) |
| Pseudo R^2^ | 0.238 | 0.238 | 0.239 |
| $\hat{P_{it}}$: Mean | 0.062 | 0.062 | 0.062 |
| Std. Dev. | 0.093 | 0.093 | 0.093 |
| Private insurance prevalence | No | Yes | Yes |
| Regional specific time trends | No | No | Yes |

^a^ The equation includes a constant term and dummy variables for gender, age, predominant diseases, hospital, area, and year.

^b^ Upper values are estimated arc elasticities, and clustering robust standard errors allowing for correlated residuals within prefectures are in parentheses.

^c^ ** and * represent statistical significance at the 1 and 5 percent levels.
